# Prevalence and course of anxiety and depression among patients selected for bariatric surgery

**DOI:** 10.1192/bjo.2021.120

**Published:** 2021-06-18

**Authors:** Jonathan Gibb, Chris Rogers, Eleanor Gidman, Graziella Mazza, Jane Blazeby, Paul Moran

**Affiliations:** 1 Centre for Academic Mental Health, Population Health Sciences, Bristol Medical School, University of Bristol; 2 Bristol Trials Centre (CTEU), Bristol Medical School, University of Bristol, Level 7, Zone A, Bristol Royal Infirmary; 3 Centre for Surgical Research, Bristol Medical School, Population Health Sciences, University of Bristol

## Abstract

**Aims:**

To determine the prevalence of anxiety and depression amongst participants with severe or complex obesity randomised and selected for bariatric surgery in a large multi-centre trial.

To describe the change in prevalence of anxiety and depression amongst participants who had undergone bariatric surgery, within 6 months of randomisation and at 12 months post-randomisation.

**Method:**

The By-Band-Sleeve (BBS) study is a multi-site randomised controlled trial evaluating the surgical management of severe or complex obesity and is the largest trial of its kind. Participants completed the Hospital Anxiety and Depression Scale (HADS) on study enrolment (pre-randomisation) and at 12 months post-randomisation. In this sub-study, we describe provisional data concerning the baseline prevalence of anxiety and depression along with change in median HADS symptom score amongst those who actually underwent bariatric surgery.

**Result:**

758 participants met the criteria for study inclusion with 716 (94.46%) and 712 (93.93%) individuals fully completing questionnaires for HADS-A and HADS-D. At pre-randomisation, the prevalence of possible (HADS A/D = 8-10) and probable (HADS A/D >11) anxiety or depression was 46.19% (n 330/716) and 48.17% (n 48.17%) respectively. Paired and complete HADS-A and HADS-D questionnaires were available for 70.25% (n 503/716) and 69.94% (n 498/712) participants. There was a highly statistically significant decrease in median HADS-A and HADS-D scores at 12 months post-randomisation (Wilcoxon signed-rank test p < 0.001). This was coupled with a statistically significant reduction in the proportion of cases with possible and probable anxiety (–9.54%, p < 0.001) and also depression (–22.21%, p < 0.001) at 12 months post-randomisation.

**Conclusion:**

Our results characterise the high rate of psychological comorbidity amongst patients with severe or complex obesity selected for bariatric surgery. Whilst bariatric surgery remains the most clinically effective treatment for severe obesity, its effects on long-term post-operative mental health outcomes are less clear. These findings contribute to the growing body of evidence calling for increased pre/post-operative mental health surveillance and integrated care for this cohort of patients.

